# Hidden Histoplasmosis: Adrenal and Central Nervous System Mycosis in an Immunocompetent Patient

**DOI:** 10.7759/cureus.90831

**Published:** 2025-08-23

**Authors:** Yi Sul Richter, Dorothy X Kenny, Sarah B Semon, Karleen M Meiklejohn, Derek J Bays, Lauren E Damon

**Affiliations:** 1 Internal Medicine, University of California, Davis, Sacramento, USA; 2 Infectious Diseases, University of California, Davis, Sacramento, USA; 3 Pathology and Laboratory Medicine, University of California, Davis, Sacramento, USA

**Keywords:** adrenal failure, adrenal histoplasmosis, cns histoplasmosis, mimic malignancy, progressive disseminated histoplasmosis

## Abstract

Histoplasmosis is a fungal infection caused by the dimorphic fungus *Histoplasma capsulatum. *Macroconidia released from hyphae growing in the soil are inhaled into the lungs, where there is a morphologic change to the pathogenic yeast form. While most infections are either asymptomatic or subclinical, histoplasmosis can present with a wide variety of clinical syndromes ranging from pneumonia to disseminated disease in virtually any organ system. Disseminated disease is typically associated with immunosuppression, specifically T-cell-mediated immunity. Here, we present a case of disseminated histoplasmosis to the central nervous system and adrenal glands complicated by adrenal crisis mimicking metastatic cancer. This occurred without traditional risk factors for disseminated histoplasmosis or travel to classically endemic areas of histoplasmosis, stressing the importance of consideration of invasive fungal infections in light of shifting endemic regions due to climate change.

## Introduction

Histoplasmosis is an infection caused by the dimorphic fungus *Histoplasma capsulatum*. This organism is found as a mold in soil, particularly soil with bat and bird droppings [1]. Infection occurs via inhalation of conidia into the lungs, where the fungus transforms into its pathogenic yeast form due to an increase in temperature [1,2]. Following replication of this form within macrophages, the organism can spread hematogenously to other organs through the reticuloendothelial system [3].

The presentation of histoplasmosis is highly variable, ranging from asymptomatic infection to life-threatening disseminated disease. Infection may be acute or slowly progressive. In most immunocompetent hosts, the immune response is sufficient such that individuals rarely become symptomatic unless they are exposed to a large inoculum of conidia [4]. Disseminated histoplasmosis is rare and typically occurs in immunocompromised patients such as those with HIV, solid organ or hematopoietic stem cell transplants, those taking immunosuppressants, or young children [4]. Patients with disseminated histoplasmosis most often present with fever, fatigue, anorexia, and weight loss and may have evidence of organ failure, shock, respiratory failure, and coagulopathy [4]. Half of patients may have hepatosplenomegaly and diffuse lymphadenopathy [4]. Adrenal gland involvement is observed in 80% of disseminated disease; however, primary adrenal insufficiency and acute adrenal crisis are rare complications, occurring in 7-20% of such cases [5]. Central nervous system (CNS) involvement is also rare, with an estimated occurrence of five to ten percent in disseminated histoplasmosis cases [6].

Here, we present a case of an immunocompetent patient with disseminated histoplasmosis involving the adrenal glands and CNS whose course was complicated by primary adrenal insufficiency and acute adrenal crisis. This atypical case highlights the challenges associated with early recognition and diagnosis of disseminated histoplasmosis in immunocompetent patients with non-specific presentations.

This article was previously presented as a meeting poster at the Society of Hospital Medicine Sacramento Chapter Scientific Poster Competition on October 12, 2024, and the American College of Physicians California Northern Region Scientific Meeting on October 26, 2024.

## Case presentation

**Figure 1:**
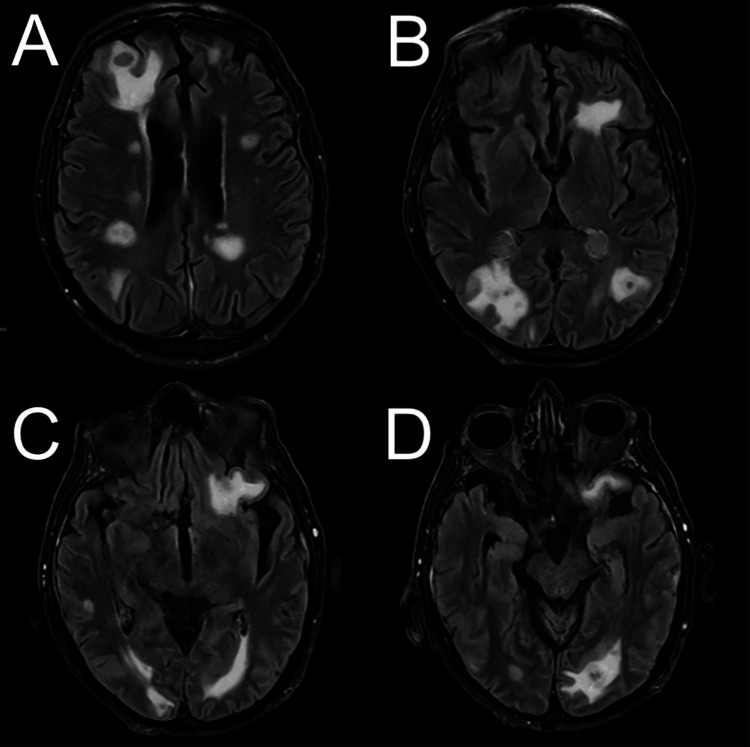
MRI of the brain with and without contrast. T2 flair images demonstrating multiple rim-enhancing lesions through selected sections from most cranial (A) to most caudal (D).

**Figure 2:**
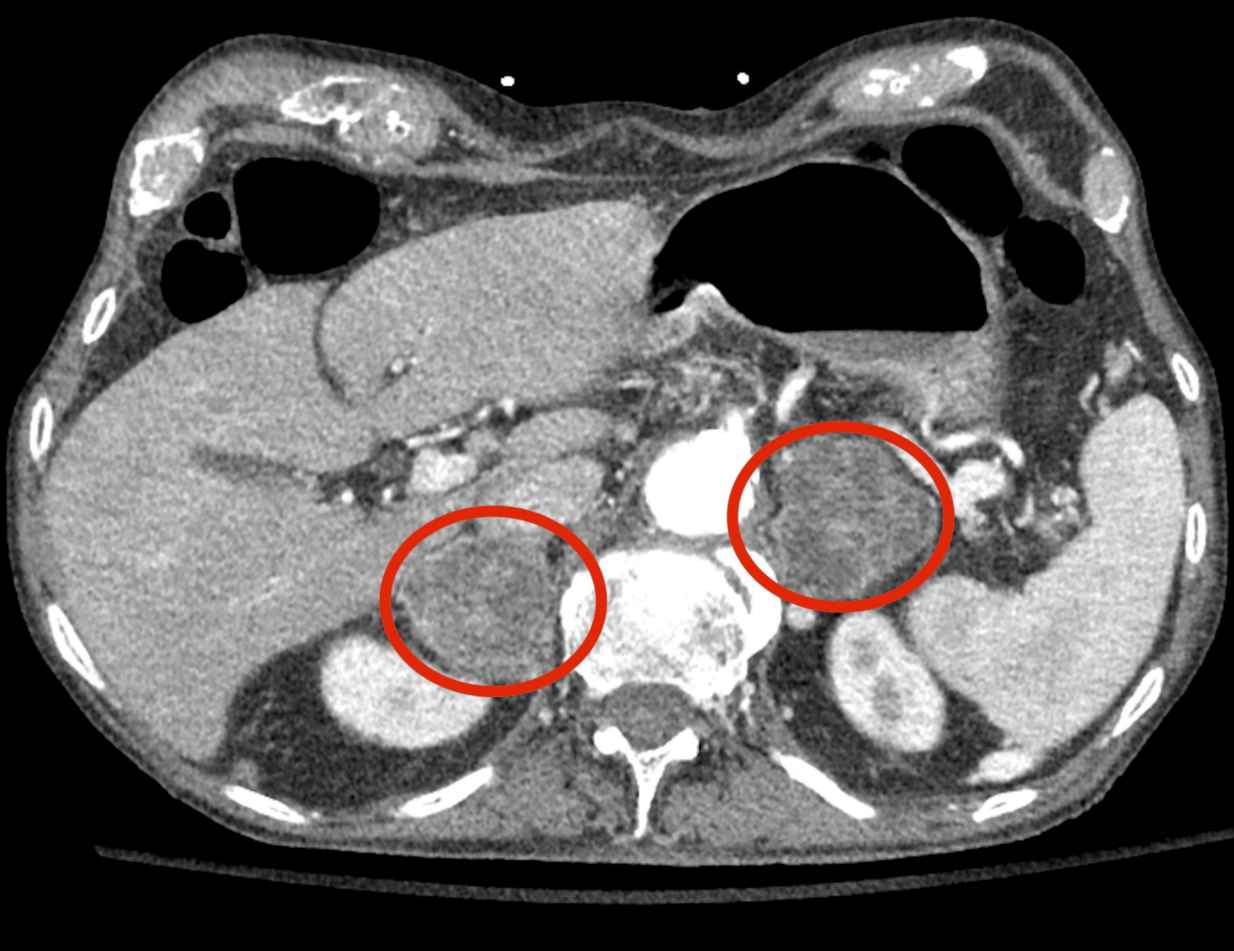
CT abdomen and pelvis with contrast demonstrating bilateral adrenal masses (red circles).

**Figure 3:**
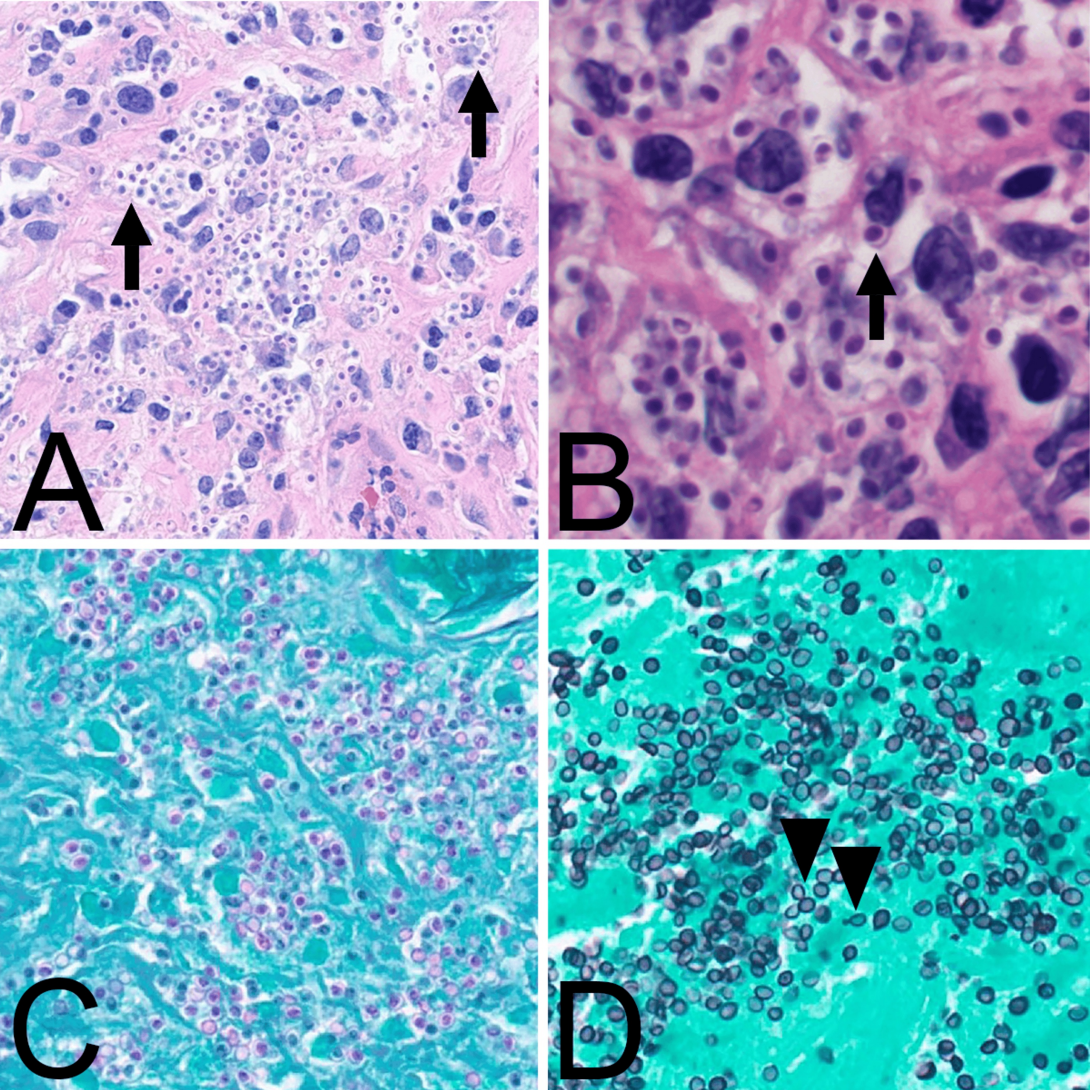
Pathology slides from adrenal biopsy. A, B: H&E-stained sections showing yeast forms with lucent "halos" (arrows) clustered in the cytoplasm of macrophages, 60x and 80x magnification, respectively. C: Periodic acid-Schiff-stained section from an area of necrosis (60x magnification). Without viable macrophages in this area, the yeast forms have spilled out of the cytoplasm and are arranged haphazardly. D: Gomori methenamine silver-stained section from an area of necrosis (60x magnification). Similar haphazard arrangement as image C. Narrow-based budding (arrowheads) is seen best here.

An 85-year-old man with a history of aortic stenosis status post-transcatheter aortic valve replacement (TAVR) presented with two weeks of progressive generalized weakness, anorexia, and acute altered mental status after a trip to Mexico. Approximately three months prior to the presentation, the patient underwent a TAVR. He did well post-procedure with improvement in functional status and subsequently traveled to Mexico, where he resided until presentation at our hospital. Vital signs were within normal limits. The exam revealed an ill-appearing, cachectic man, only oriented to self without any focal neurologic deficits. The workup was notable for a new elevation of liver function tests with an aspartate aminotransferase (AST) of 70 U/L, alkaline phosphatase of 152 IU/L, total bilirubin of 1.5 mg/dL, lactate dehydrogenase of 288 U/L, and thrombocytopenia of 141,000 cells/µL. Other pertinent results included a sodium of 139 mmol/L, potassium of 5.1 mmol/L, glucose of 95 mg/dL, and a white blood cell count of 5,600 cells/µL, all within normal limits (Table 1). MRI of the brain demonstrated multiple ring-enhancing lesions initially concerning for metastatic disease with unknown primary (Figure 1). A CT of the chest, abdomen, and pelvis demonstrated new bilateral adrenal masses (Figure 2). Given concern for malignancy, cerebral spinal fluid studies were limited to mostly non-infectious studies (Table 2); however, fungal cultures were negative. A brain biopsy was considered; however, an adrenal biopsy was pursued to exhaust less invasive diagnostic opportunities. Pathology revealed necrotic tissue, which prompted additional staining for infections, identifying intracellular organisms consistent with *Histoplasma* (Figure 3). Serum *Histoplasma* antibody (ARUP Laboratories, Salt Lake City, Utah, USA) was positive with an M band, suggesting recent infection. *Histoplasma* Galactomannan Antigen Quantitative by Enzyme Immunoassay (EIA) from the urine (ARUP Laboratories) was negative, and *Histoplasma* Galactomannan Antigen Quantitative by EIA from the serum (ARUP Laboratories) was indeterminate (indicating questionable presence of *Histoplasma* antigen) (Table 3). Brain biopsy was again considered to confirm the diagnosis of disseminated histoplasmosis, given the discordant noninvasive test results, but was deferred given the patient’s clinical status and pathologic findings. The patient was started on intravenous liposomal amphotericin B with a slight improvement in the size of CNS lesions after one week. He later required transfer to the intensive care unit due to the development of shock, acute kidney injury, and progressive altered level of consciousness. Stress-dose hydrocortisone was given for suspected adrenal crisis with improvement in hemodynamic stability. A cosyntropin stimulation test confirmed adrenal insufficiency. Despite maximum medical therapy, the patient continued to worsen and was ultimately discharged under hospice care.

**Table 1:** Laboratory findings on initial presentation.

Parameter	Result	Reference Range
Serum Sodium	139 mmol/L	136 - 145 mmol/L
Serum Potassium	5.1 mmol/L	3.4 - 5.1 mmol/L
Aspartate Aminotransferase	70 U/L	= 41 U/l
Alkaline Phosphatase	152 U/L	35 - 129 U/L
Total Bilirubin	1.5 mg/dL	= 1.2 mg/dL
Lactate Dehydrogenase	288 U/L	135 - 225 U/L
White Blood Cell Count	5,600 cells/µL	4,500 - 11,000 cells/µL
Platelets	141,000 cells/µL	130,000 - 400,000 cells/µL
Glucose	95 mg/dL	74 - 109 mg/dL

**Table 2:** Cerebral spinal fluid analysis from serial lumbar punctures.

Parameter	Lumbar Puncture 1	Lumbar Puncture 2	Reference Range
Total Nucleated Cells	1 cell/µL	3 cells/µL	= 5 cells/µL
Red Blood Cells	7 cells/µL	106 cells/µL	0 cells/µL
Glucose	47 mg/dL	Not tested	40 - 70 mg/dL
Protein	55 mg/dL	Not tested	15 - 45 mg/dL
Bacterial Culture	No growth	Not tested	No growth
Fungal Culture	No growth	Not tested	No growth
Meningitis/Encephalitis Panel	Negative	Not tested	Negative
Flow Cytometry	No monotypic B cell population	No monotypic B cell population	-

**Table 3:** Relevant microbiologic studies. PCR: polymerase chain reaction; EIA: enzyme immunoassay

Parameter	Result	Reference Range
*Histoplasma* Antigen Quantitative by EIA, Serum	Indeterminate	Not detected
*Histoplasma* Galactomannan Antibody, Urine	Not detected	Not detected
*Histoplasma* Antibodies by Complement Fixation and Diffusion, Serum	Detected	Not detected
Toxoplasma Antibody, IgG, Serum	Positive	Negative
Toxoplasma Gondii Antibody, IgM Serum	<3.0 AU/mL	= 7.9 AU/mL
Toxoplasma Gondii by PCR, Serum	Not detected	Not detected
Coccidiodes Immunodiffusion	Equivocal IgM	Not detected
Coccidiodes Complement fixation	Negative	Negative

## Discussion

The severity of disease in patients with histoplasmosis is largely determined by the amount of conidia inhaled and the characteristics of the host’s immune system [7]. Immunocompetent hosts that inhale a small amount of conidia typically have asymptomatic or mild disease, but severe disease can occur with larger inoculum exposure [7]. Acute pulmonary histoplasmosis is often self-limited and most commonly occurs in children after first-time exposure to the organism [3]. Clearance of the yeast is primarily achieved via CD4+ T cell-mediated immunity; therefore, compromise of this response can result in disseminated disease [7]. Consequently, patients with HIV/AIDS, certain hematologic malignancies, congenital T cell deficiencies, solid organ or hematopoietic stem cell transplant recipients, and those taking immunosuppressive agents such as TNF alpha inhibitors or corticosteroids are at increased risk of developing disseminated disease and mortality [3,8]. Extremes of age have also been characterized as a risk factor for disseminated histoplasmosis. In this case presentation, the patient’s only identifiable risk factor was advanced age [4].

This case demonstrates a rare occurrence of disseminated histoplasmosis involving both the adrenal glands and CNS. A prior study found that the adrenal glands were involved to some extent in 23 of 28 (82%) autopsies of patients with disseminated histoplasmosis, which suggests that adrenal gland involvement may be common in disseminated disease [9]. However, of the 28 autopsy cases, only three cases of primary adrenal insufficiency (11%) were identified, and across the entire case series of 84 patients with disseminated histoplasmosis, primary adrenal insufficiency was identified only six times (seven percent) [9]. Acute adrenal crisis secondary to primary adrenal insufficiency is only described in a few cases of disseminated histoplasmosis, further demonstrating the atypical nature of this case [10]. The differential diagnosis for bilateral adrenal lesions is broad, including etiologies such as malignancy, trauma, autoimmune adrenalitis, and infections such as tuberculosis, histoplasmosis, blastomycosis, and parasitic cysts [11,12]. Considering the patient’s non-specific presentation, unrevealing infectious workup early in the course, and the lack of typical risk factors for the aforementioned infectious processes, malignancy was higher on the differential, which delayed further infectious workup and, consequently, the diagnosis and treatment of disseminated histoplasmosis. While infections are rarer causes for bilateral adrenal lesions, suspicion should increase when pathologic examination demonstrates predominantly necrosis, granulomatous inflammation, or microorganisms.

Another factor complicating this patient’s hospital course was the presence of multiple ring-enhancing lesions on brain MRI that were initially suspected to be metastatic malignancy. CNS involvement is estimated to occur in five to ten percent of patients with disseminated histoplasmosis and is more common in immunocompromised patients [6]. Dissemination to the CNS is associated with a mortality rate of 20-40% and a relapse rate of 50% [13]. Patients often present with indeterminate symptoms such as altered mental status and generalized weakness, as in this case, making diagnosis a challenge [6]. Indeed, case reports reveal that diagnosis is frequently delayed, particularly in immunocompetent patients presenting with symptoms of chronic meningitis [4]. Given its non-specific presentation, disseminated histoplasmosis to the CNS has been misdiagnosed as primary hematologic malignancy, tuberculosis, neurosarcoidosis, toxoplasmosis, multiple sclerosis, and Behcet’s syndrome [6,14].

Clinical evaluation of disseminated histoplasmosis poses several difficulties, particularly in patients lacking classic risk factors. First, histoplasmosis is less likely to be suspected in patients from non-endemic areas [15]. Histoplasmosis is historically endemic to South America, North America (particularly the Mississippi and Ohio River Valleys), and sub-Saharan Africa [16]. However, in the setting of climate-related changes, histoplasmosis is becoming more common in a larger geographic area, and outbreaks have been observed in more urban rather than rural areas [16]. Furthermore, there is concern that elevated temperatures and increased ultraviolet radiation exposure may result in more pathogenic strains [16]. In this case, given the lack of other travel history, our patient was most likely exposed to histoplasmosis during his recent trip to Mexico.

Histoplasmosis is also less likely to be considered in immunocompetent patients, which may further delay diagnosis. The differential diagnosis for patients presenting with altered mental status, malaise, anorexia, and weight loss is broad and difficult to narrow with a standard workup. In this case, initial CSF studies were consistent with aseptic meningitis, and the brain lesions were suspected to be metastases from an unidentified primary malignancy. The adrenal masses were thought to be incidentalomas, and lab work early in the patient’s course was not indicative of overt primary adrenal insufficiency. Considering the rarity of disseminated histoplasmosis, particularly involving the adrenal glands and/or CNS in immunocompetent patients, further infectious workup was delayed until histopathology of an adrenal gland biopsy was suggestive of the diagnosis.

Typically, antigen and antibody testing are utilized in the initial evaluation for histoplasmosis, given their non-invasive nature and rapid results. However, the sensitivity and specificity of urine and serum antigen testing vary in localized versus disseminated disease [17]. The sensitivity of the urine antigen test is superior to that of the serum antigen test in disseminated disease but not in localized pulmonary infection [17]. False negative results for antigen tests are more likely in localized pulmonary histoplasmosis compared to disseminated disease [18]. This case was unique in that the urine antigen was falsely negative and the serum antigen was indeterminate despite disseminated disease. Antigen detection in CSF may be used to diagnose disseminated histoplasmosis involving the CNS; however, sensitivity ranges from 40 to 65%, and this test was not done in this case, given the histopathologic findings obtained from the adrenal gland biopsy [19]. The gold standard for diagnosis of histoplasmosis is isolation of the organism in culture or histopathology, which necessitates an invasive procedure, and results may take two to six weeks. This case demonstrates the importance of obtaining histopathology and/or culture, given the potential for falsely negative antigen and antibody testing. Ideally, culture or histopathology should be pursued early in the patient’s course, given the additional time required for coordination and processing. This emphasizes the need for early consideration of disseminated histoplasmosis and broadening of diagnostics as the geographic distribution of infectious diseases and the profile of susceptible hosts continue to evolve [20].

## Conclusions

While disseminated histoplasmosis is rare and classically associated with immunocompromising conditions, this case demonstrates the need to consider this increasingly endemic infection even without traditional risk factors. Adrenal involvement can precipitate primary adrenal insufficiency and acute adrenal crisis, while CNS disease carries a higher risk of morbidity and mortality. Bilateral adrenal involvement, especially without a discrete mass and evidence of necrosis on pathology, should heighten suspicion for infectious etiologies. Diagnosis of disseminated histoplasmosis remains a clinical challenge, as patients frequently present with non-specific symptomatology, and the initial diagnostic workup may be unrevealing, which can delay treatment and worsen outcomes. In patients presenting with malignant-appearing disease, clinicians should avoid premature closure and consider infectious etiologies, especially in patients returning from areas endemic for infections or who have necrosis on biopsy findings.
